# Chloralum

**Published:** 1871-08

**Authors:** 


### Chloralum.

Dr. Edward Ballard, Medical Officer of Health for Islington (*Chemical News*, January 20, 1871), protests against the assertion made by Professor Gamgee, that carbolic acid, owing to its smell, is less used than it would be if without odor, and recommends caution in accepting chloralum as a disinfectant. In his own experience he has found carbolic acid "a most efficient agent for destroying contagia," and that its odor is not offensive unless contaminated with sulphide of ammonium.

He objects to the inference that because chloralum is *antiseptic* it is also *disinfectant*, and to Professor Gamgee's assuming its disinfecting power from its chemical properties as an antiseptic. A disinfectant, he says, is an agent which will destroy the vitality—the power of growth and reproduction—of most minute particles of matter which, given off by the sick, are capable of producing a like disease in the healthy. He thinks that to prove that any substance is a disinfectant, it should be shown by experiment, by an accomplished microscopist, to have the power of destroying the vital manifestations of those minute amœbiform particles of matter which constitute the simplest form of living things, and by repeated experiment upon a large scale, that the repeated use of the disinfectant has actually resulted in the arrest and spread of contagious disease. He is of the opinion that Prof. Gamgee has advanced nothing to satisfy any one that chloralum, used in any way, is capable of destroying the peculiar manifestations of a morbid contagion, and thinks that the reason for its rapid strides into the favor of medical men (who are apt to take up new disinfectants in a "wild manner") may be found in that freedom from odor which Prof. Gamgee considers the basis on which the reputation of Condy's fluids rest.

Dr. Ballard gives as a reason for not having the chloralum himself, though small-pox and scarlet fever are raging in his district, that he dare not assume the responsibility of its use until *prima fàcie* proof at least is afforded him that by using it he will be using that which is capable of destroying "disease-germs."

In the same journal (January 27, 1871), Prof. Gamgee expresses his high appreciation of the value of the suggestions made by Dr. Ballard with regard to means for investigating and proving the mode of action of substances offered as disinfectants, but thinks that little would be learned experimentally about any of them if all persons who, like Dr. Ballard, have abundant oppo rtunity of testing the matter, waited instead of acting.

He states that chloralum shrivels, arrests the movements of, and kills the amœbiform bodies referred to,—and does more, it destroys many of the lower forms of parisitic life, whether animal or vegetable. He is convinced that every good antiseptic is really a destroyer of disease. He adds that the properties of chloralum are almost identical with the active antiseptic and disinfectant properties of hydrochloric acid.—*New Remedies.*

